# Short treatment with antalarmin alters adrenal gland receptors in the rat model of endometriosis

**DOI:** 10.1371/journal.pone.0227456

**Published:** 2020-01-14

**Authors:** Annelyn Torres-Reverón, Maahrose Rana, Varesh Gorabi, Leslie L. Rivera-Lopez, Caroline B. Appleyard

**Affiliations:** 1 DHR Health Institute for Research and Development, Edinburg, Texas, United States of America; 2 Dept. of Biomedical Sciences, University of Texas at Rio Grande Valley, Edinburg, Texas, United States of America; 3 Dept. Neuroscience, University of Texas at Rio Grande Valley School of Medicine, Edinburg, Texas, United States of America; 4 Division of Basic Sciences, Ponce Health Sciences University—Ponce Research Institute, Ponce, Puerto Rico, United States of America; 5 Dept. of Internal Medicine, Ponce Health Sciences University, School of Medicine, Ponce, Puerto Rico, United States of America; University of Insubria, ITALY

## Abstract

Endometriosis is a chronic inflammatory disorder in which endometrial tissue is found outside the uterine cavity. Previous reports suggest that there is a dysregulation of the hypothalamic pituitary adrenal axis during the progression of endometriosis. Our previous report showed that a short-term treatment with antalarmin, a corticotrophin releasing hormone receptor type 1 (CRHR1) antagonist decreases the number and size of endometriotic vesicles in the auto-transplantation rat model of endometriosis. Our current goal was to examine the mRNA expression of intra-adrenal receptors to better understand the mechanisms of the hypothalamic pituitary adrenal (HPA) axis involvement in endometriosis. We used two groups of female rats. The first received sham surgery or endometriosis surgery before collecting the adrenals after 7 days of the disease progression. The second group of animals received endometriosis surgery and a treatment of either vehicle or antalarmin (20 mg/kg, i.p.) during the first 7 days after endometriosis induction and then the disease was allowed to progress until day 60. Rats with sham surgery served as controls. Results showed that the mRNA expression of the mineralocorticoid (MRC2) receptor was lower in the rats after 7 days of endometriosis surgery and in rats with endometriosis that received antalarmin. In addition, the CRHR1 was significantly elevated in animals that received antalarmin and this was counteracted by a non-significant elevation in CRHR2 mRNA. The glucocorticoid receptor mRNA within the adrenals was not affected by endometriosis or antalarmin treatment. This report is one of the first to explore intra-adrenal mRNA for receptors involved in the HPA axis signaling as well as in the sympatho-adrenal signaling, calling for additional research towards understanding the role of the adrenal glands in chronic inflammatory diseases such as endometriosis.

## Introduction

The adrenal glands are triangular shaped organs that sit on top of the kidneys, each composed of an outer cortex and an inner medulla. While the medulla mainly secretes the catecholamines epinephrine and norepinephrine, the cortex is subsequently subdivided into three zones: the glomerulosa, secreting aldosterone, pregnenolone and progesterone; the fasciculata, secreting cortisol and corticosterone and the reticularis, secreting dehydroepiandrosterone (DHEA) and androstenedione [1]. The adrenals are the last effector in the hypothalamic pituitary adrenal (HPA) axis. In response to stress, corticotropin releasing hormone (CRH) is synthesized in the hypothalamus and travels to the anterior pituitary, where it binds to corticotropin releasing hormone receptors (CRHR), stimulating the production of adrenocorticotropic hormone (ACTH). ACTH is released into the blood and through binding specifically with the mineralocorticoid 2 receptor (MCR2) mainly in the zona fasciculata of the adrenal cortex, results in the synthesis and secretion of corticosterone and cortisol [2,3]

Adrenocortical hormone secretion, specifically corticosterone (CORT) and cortisol, are tightly regulated via negative feedback mechanisms. Glucocorticoid receptors (GR) are expressed in the anterior pituitary but are also present in the adrenal cortex [4]. In addition to GR-mediated ACTH regulation at the level of the pituitary [5], there is evidence supporting an intra-adrenal negative feedback loop where increases in local CORT concentration act to down-regulate the production of more glucocorticoids, via GR located on the adrenal glands [6]. Furthermore, Walker and colleagues also found in adrenal cells that adding higher concentrations of CORT inhibited its own synthesis most likely via a non-genomic intra adrenal pathway involving GR [6]. CORT is not stored in the adrenals, but rather released into the circulation upon synthesis. Corticosterone binding globulin (CBG) binds to CORT and regulates target tissues and inflammatory sites. CBG not only limits CORT, but also protects glucocorticoids from degradation and maintains an equilibrium of plasma glucocorticoid levels for the target tissue [7]. There is also basis for a CRH regulatory system in the adrenals. In a mouse model study, it was suggested that the CRH receptor type 1 (CRHR1) in the adrenal cortex is involved in the release of corticosterone, acting separately from the pituitary [8]. CRH receptors have been reported in both the cortex and medulla of human, bovine, canine and rodent adrenal glands [9–15] alluding to a highly-regulated intra adrenal signaling system.

Prolonged stress caused by chronic disease progression can dysregulate this HPA axis [16]. For example, women with endometriosis present lower levels of serum cortisol [17,18] although high cortisol levels have also been reported in hair samples [19]. This discrepancy in cortisol levels evaluated in women with endometriosis could be due to numerous factors which possibly include: the type of tissue used to evaluate the levels of cortisol, age of the individual, and season of the year when sample was collected [20]. However, whether the changes in cortisol due to endometriosis is produced as a response to the physical and emotional stress caused by the disease or it is an ethiological characteristic of the disease, still needs to be elucidated. In other inflammatory disorders which cause pelvic pain and inflammation such as irritable bowel syndrome, abnormal adrenal activity has been reported by assessment of DHEA and cortisol [21]. Therefore, due to the variability that cortisol levels might produce across studies, examining the adrenal activity directly might provide better evidence for understanding the role of the HPA axis in endometriosis progression and other inflammatory disorders.

Previously, our group documented the effects of systemic administration of antalarmin, a non-peptide CRHR1 antagonist with high affinity for the receptor (Ki = 0.8 nM). This drug belongs to the family of pyrrolopyrimidine derivatives and readily crosses the blood brain barrier. Antalarmin can lessen the severity of endometriosis in the auto-transplantation rat model, but caused a prolonged increase in ACTH levels [22]. In light of clinical and animal data suggesting a sustained de-regulation of the HPA axis, here we quantified the mRNA expression of intra-adrenal receptors: MCR2, GR, CRHR1 as well as CRHR2. By focusing on the expression of the adrenal receptors we can infer adrenal activity under a chronic inflammatory condition such as endometriosis, and consequences after administration of a CRHR1 antagonist. We hypothesized that antalarmin will reverse any effects on CRHR1 within the adrenals, but other receptors will show normal mRNA levels. Our results demonstrate a general deregulation of MC2R as well as CRHR1 in the adrenal glands during endometriosis and administration of antalarmin. This is the very first report to focus on adrenal activity during endometriosis progression.

## Materials and methods

### Animals and endometriosis induction

Adrenal tissue was collected from experimental animals used in our prior published experiments [22]. Experimental procedures were previously approved by the Ponce Health Sciences University (protocol #202) and the University of Texas at Rio Grande Valley (protocol #2016–004) Institutional Animal Care and Use Committees and adhere to the NIH Guide for the Care and Use of Laboratory Animals.

**Fig 1:**
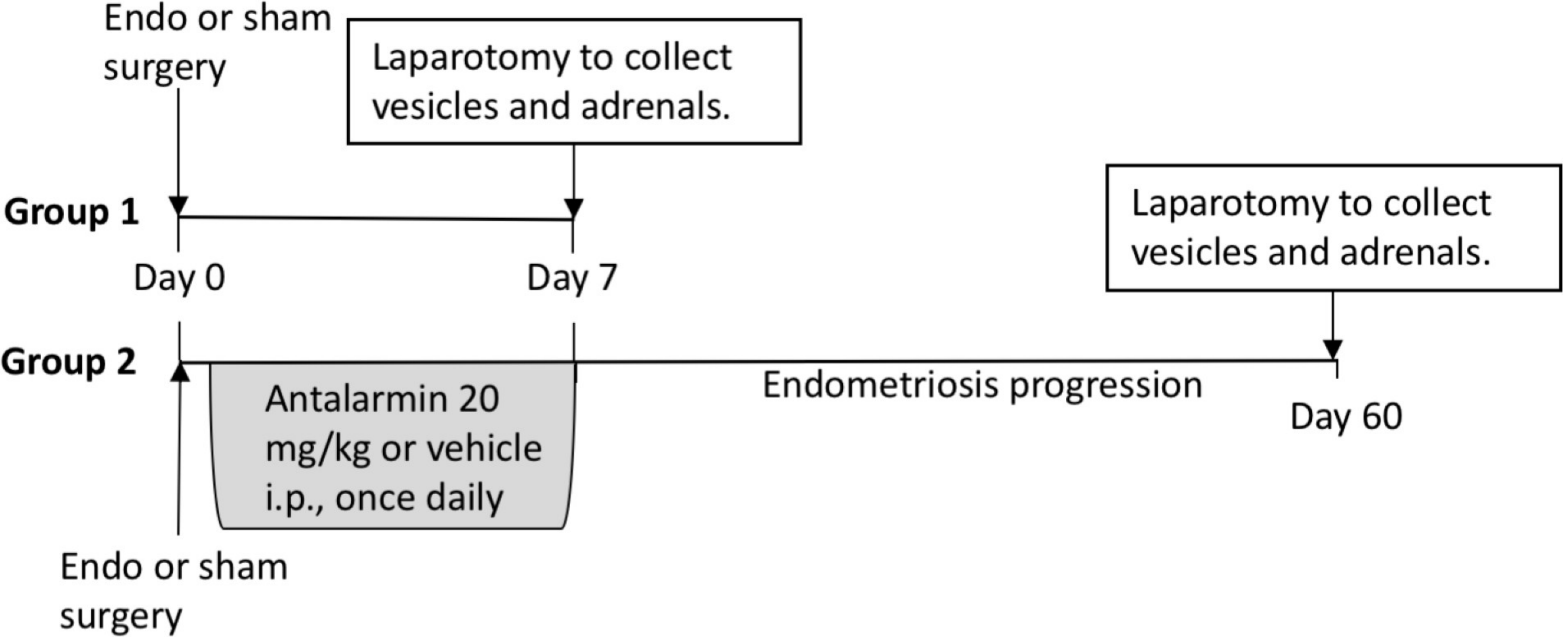
Diagram of experimental protocols. The protocol for tissue collection used in the current manuscript is similar to the one previously published [22]. Two separate cohorts of rats were used: one that received endometriosis or sham surgery and allowed to progress until 7 days after surgery, and one that received antalarmin or vehicle with endometriosis surgery which was allowed to progress until day 60. Sham rats in group 2 did not receive any treatment and were used for baseline comparisons.

For the surgical induction of endometriosis, rats were anesthetized with isoflurane and four pieces of the right uterine horn were auto transplanted to 4 different blood vessels in the intestinal mesentery. In the sham group, the right uterine horn was massaged for 2 minutes and sutures were placed in the intestinal mesenteric area with no uterine implants. We used absorbable sutures to close the muscle wall and wound clips to close the skin. Rats recovered in individual cages until fully awake and locomotive upon which they were returned to a clean home cage with their respective cage mate. Fig 1 illustrates the experimental protocols used as described below:

For Group 1 in this manuscript, adult female rats underwent endometriosis (endo-7days, n = 8) or sham surgery (sham-7days, n = 8) and were sacrificed 7 days after the surgery.

For Group 2, adult female rats underwent endometriosis surgery (endo; n = 24) or sham surgery (n = 11). The rats in the endo groups received 1 daily i.p. injection (between 09:00–10:00 hours) for 7 consecutive days of vehicle composed of 10% Tween 80 diluted with distilled water (n = 12) or 20 mg/kg of antalarmin (N-butyl-N-ethyl-[2,5,6-trimethyl-7-(2,4,6-trimethylphenyl)-7H-pyrrolo[2,3-d]pyrimidin4-yl]-amine; Tocris Bioscience, Bristol, UK) in a volume of 1 ml/kg (n = 12). Rats in the sham surgery group received nothing. Endometriosis and sham groups were sacrificed on day 60 after endometriosis induction surgery. While the number of animals per group in our previous publication [22] was larger, here we are reporting the adrenal weight, ACTH and corticosterone of a subset of only 12 rats in the endo-vehicle, 12 rats in the endo-antalarmin and 11 rats in the sham group. Rats weights, estrous cycle distribution, were previously reported [22].

### RNA isolation, cDNA synthesis and qRT-PCR

All protocols were conducted as previously described [22] using the same chemicals. The following running protocol was used for cDNA synthesis: 25°C for 5 min, 46°C for 20 min, 95°C for 1 min. For qRT-PCR cycles the protocol was as follows: 95°C for 10 min. for enzyme activation followed by 40 cycles of denaturing at 95°C for 15 sec. and annealing at 60°C for 1 min. All samples were run in duplicates and normalized against GAPDH. For standardization purposes, samples from all three groups were always run at the same time, within the same PCR plate and its respective GAPDH from each animal.

All data was analyzed using a Student t-test (Group 1) or One way-ANOVA (Group 2) within GraphPad Prism 7.0 (Graph-Pad Software, San Diego, California). Data is presented as mean difference ±SEM statistical significance was set at p <0.05.

## Results

### Group 1

**Fig 2:**
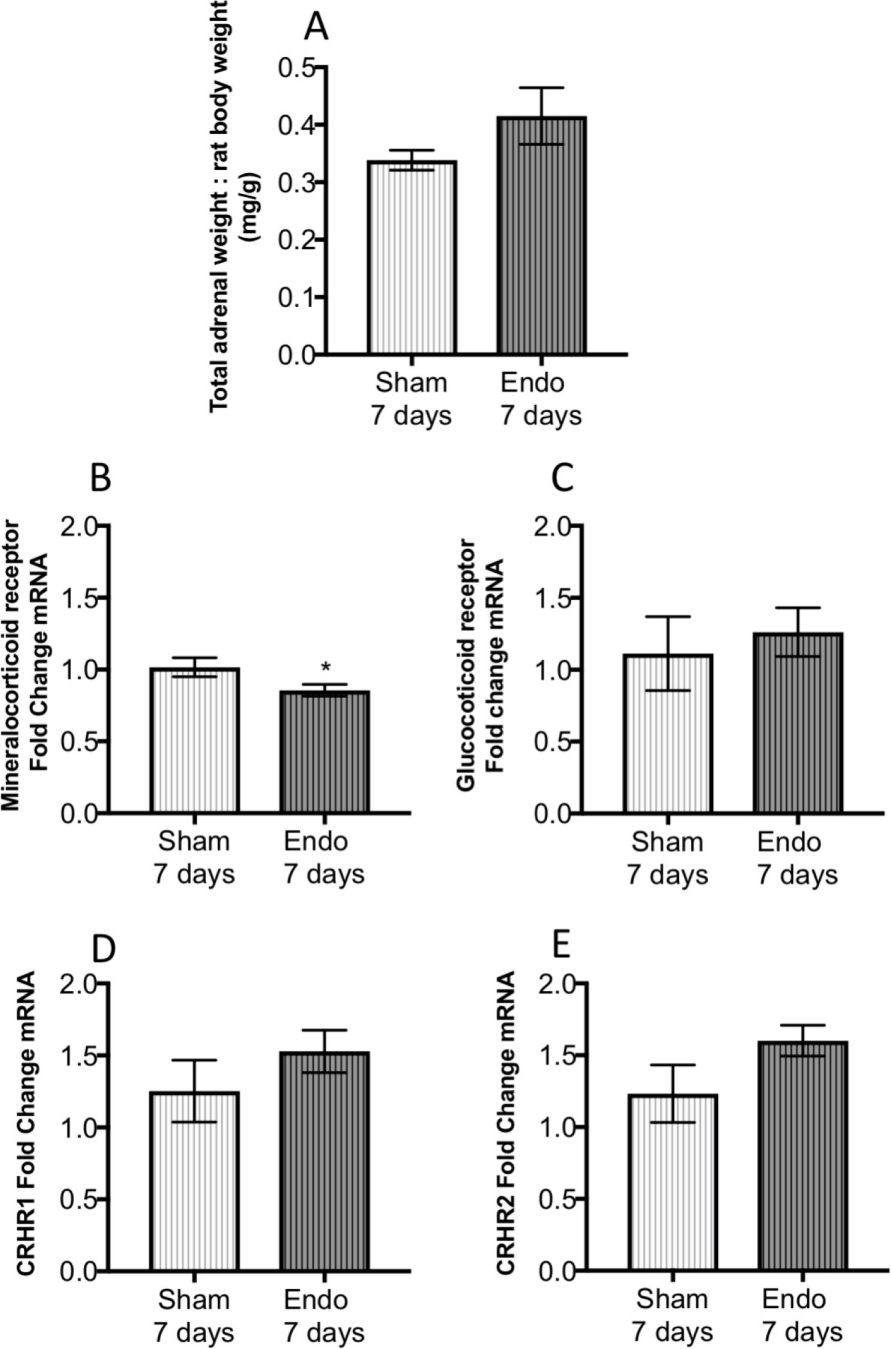
Total adrenal weight (A) and mRNA quantification of the mineralocorticoid receptor (B), glucocorticoid receptor (C), corticotrophin releasing hormone receptor type 1 (CRHR1; D) and type 2 (CRHR2; E). N = 8 in each group. For this and all subsequent panels we illustrate mean and bars represent S.E.M. * represents p< 0.05.

Seven days after endometriosis or sham surgery rats were sacrificed. Open field and zero maze behavior were collected the same morning and the behavioral results have been previously published [22]. The total adrenal weight normalized against the rat body weight at sacrifice showed a slight tendency for the weight of endo-7days to be higher, but this apparent increase was not significantly different (t = 1.74, d.f. = 14, p>0.05; Fig 2A). ACTH and corticosterone levels in the animals used herein for adrenal assessment have been previously reported [22] showing no difference between sham and endo-7 days group. Quantification of adrenal mRNA expression for the MCR2 showed a small but significant decrease in the endo-7days group compared to sham (t = 2.076, d.f. = 14, p = 0.05; Fig 2B). On the other hand, no significant changes between sham and endo-7 days in mRNA were observed for GR, CRHR1 and CRHR2 (Fig 2C, 2D and 2E).

### Group 2

Sixty days after endometriosis or sham induction surgery, animals were sacrificed. Open field and zero maze behaviors were collected on the same morning of sacrifice and these behavioral data have been shown previously [22]. Vesicle weight and size from the animals with endometriosis for which we analyzed the adrenals are detailed in Table 1.

**Table 1:** Endometriosis vesicle measurements in animals for which adrenal receptors were analyzed.

Endo vesicles at 60 days Average per rat	Percent developed (S.E.M.)	Total weight in grams (S.E.M.)	Total area in mm^2^ (S.E.M.)
Vehicle, n = 12	91.67 (3.55)	0.68 (0.20)	75.28 (13.51)
Antalarmin, n = 12	58.33 (9.89)	0.18 (0.05)	25.47 (7.64)

**Fig 3:**
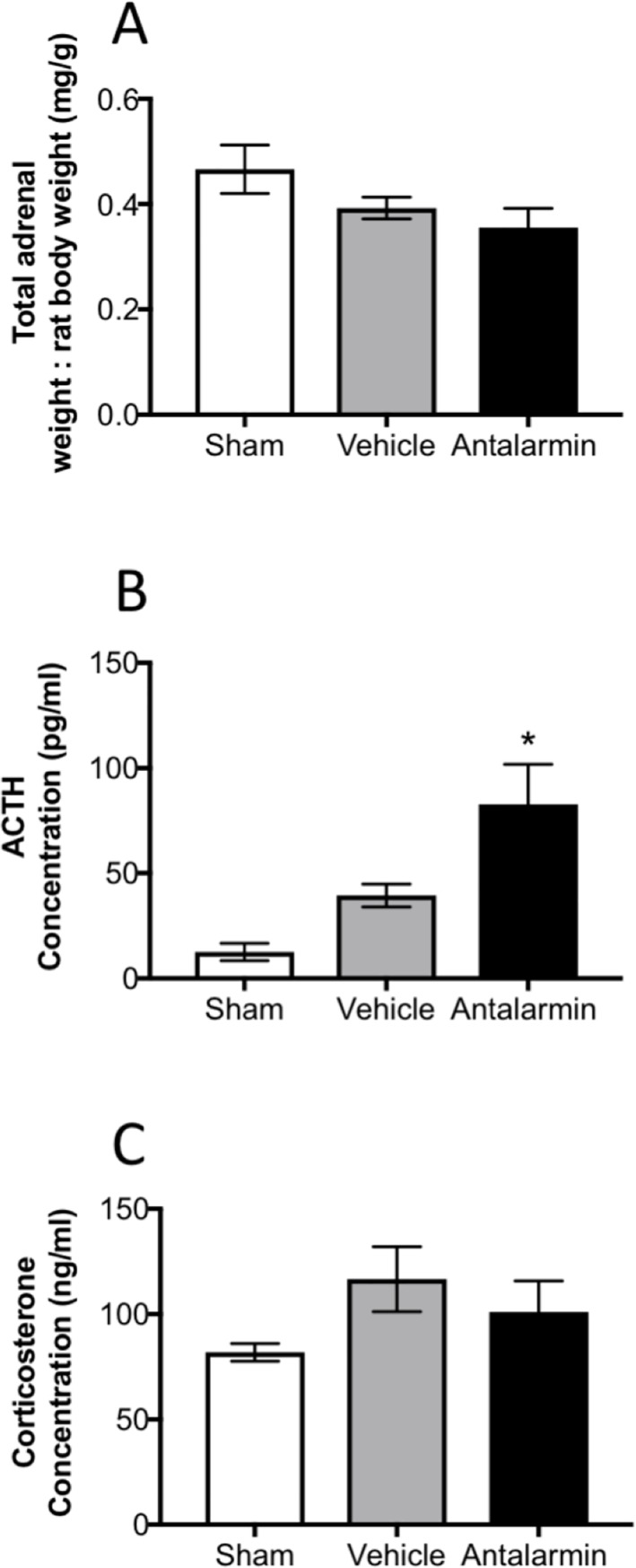
Total adrenal weight normalized by rat body weight (A), adrenocorticotropic hormone (ACTH) concentration in serum (B) and corticosterone concentration in serum (C). * represents p< 0.05 on an ANOVA test.

We also collected blood for analyzing ACTH and corticosterone in serum as well as removed and weighed both adrenal glands and normalized to total body weight of the animal at the time of sacrifice. We are only reporting ACTH and corticosterone for the animals in which adrenal mRNA was examined in this publication. No difference in adrenal weight was noted between groups although the rats with endometriosis that received antalarmin had a small tendency to be lower than the sham group but not significant (F_(2,32)_ = 2.52, P> 0.05; Fig 3A). ACTH serum concentration was elevated in the rats from the endometriosis group that received antalarmin compared to rats that received vehicle (F_(2,30)_ = 7.57, p< 0.01; Fig 3B). Corticosterone concentration in serum was similar for both groups of rats with endometriosis, regardless of treatment (vehicle or antalarmin) and these were not different from the sham group (F_(2,29)_ = 1.37, p> 0.05; Fig 3C).

**Fig 4:**
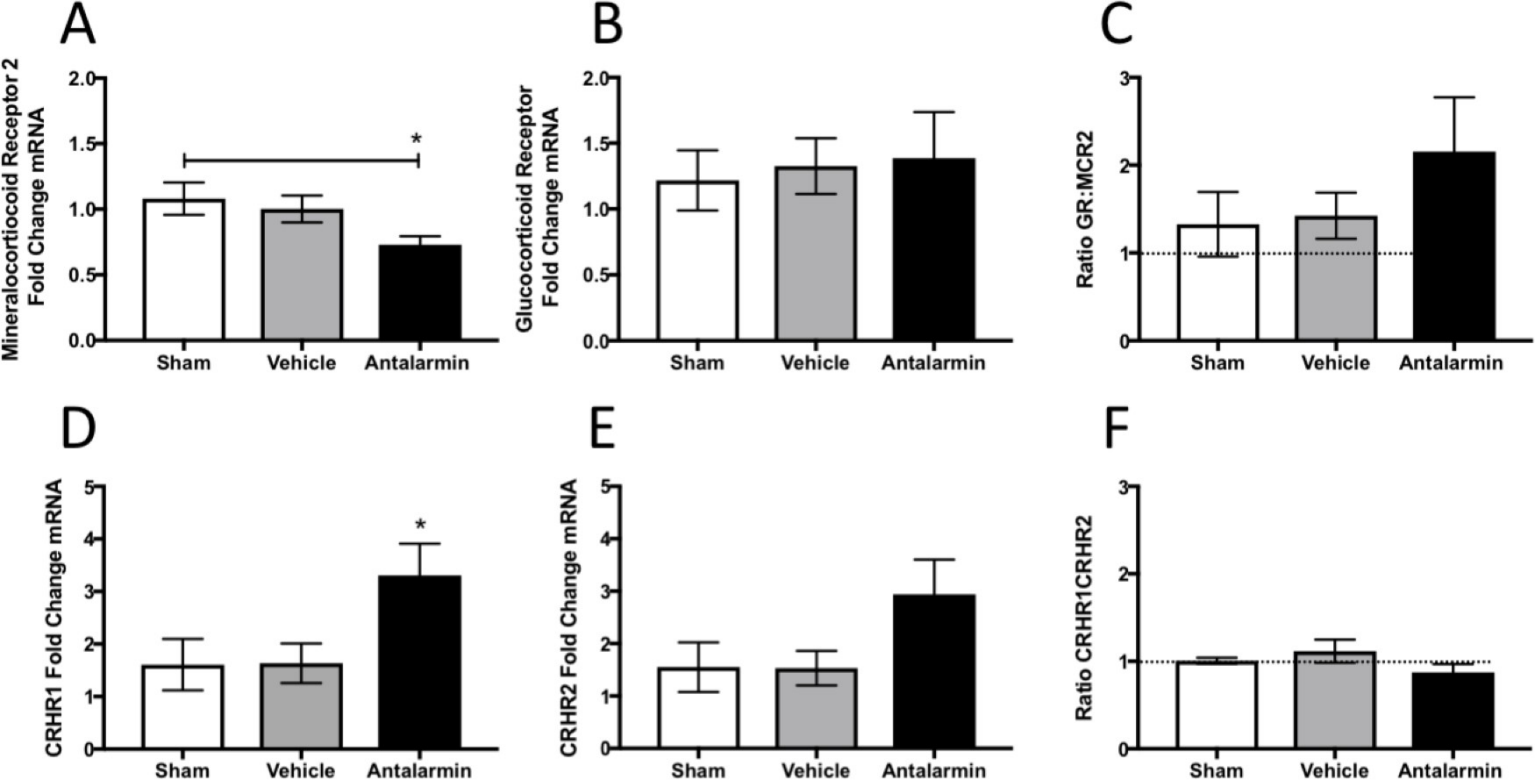
mRNA quantification of the mineralocorticoid receptor (MCR2; A), glucocorticoid receptor (GR; B). Ratio of glucocorticoid to mineralocorticoid receptors mRNA expression within the adrenals (C). mRNA quantification of the corticotrophin releasing hormone receptor type 1 (CRHR1; D) and type 2 (CRHR2; E). Ratio of CRHR1 to CRHR2 mRNA expression within the adrenals (F) * represents p< 0.05 on an ANOVA test. Dotted line represents a perfect ratio of 1:1 between receptors expression.

We examined MCR2, CRHR1 and CRHR2 mRNA within samples of whole adrenal homogenates. mRNA levels of MCR2 showed a significant difference between groups (F_(2,32)_ = 3.53, p<0.05). Pre-planned post hoc analyses revealed that antalarmin administration from days 1–7 after endometriosis surgery produced a long-lasting reduction in MCR2 mRNA compared to sham control group (p<0.05; Fig 4A). The glucocorticoid receptor mRNA did not show any difference between groups either by treatment with antalarmin or endometriosis status (F_(2,32)_ = 0.093, p> 0.05; Fig 4B). Examining the ratio of GR to MCR2 expression in the adrenals revealed that in the rats that received antalarmin and endometriosis, there is a shift towards GR expression and the ratio in this animals is 2:1 compared to the vehicle and sham operated rats, which demonstrated a ratio of GR to MCR2 closer to 1:1 (Fig 4C).

Rats with endometriosis that received vehicle showed no change compared to shams in the CRHR1 but this receptor was elevated in the rats with endometriosis that received antalarmin (F_(2,32)_ = 3.83, p< 0.05; Fig 4D). Post hoc analyses revealed that for CRHR1 mRNA, the endometriosis group treated with antalarmin was significantly higher than the sham (p<0.05) and the endometriosis group treated with vehicle (p<0.05). While qualitative observations show a similar pattern of mRNA for CRHR2, where the groups with endometriosis that received antalarmin appears elevated compared to the other two groups, this apparent difference did not reach statistical significance (F_(2,32)_ = 2.55, p = 0.09; Fig 4E). Examining the ratio of CRHR1 to CRHR2 receptor expression we can observe that all three groups showed a ratio of expression that was very close to 1:1 despite the significant increase in CRHR1 observed for rats that received antalarmin (Fig 4F).

## Discussion

We report for the first time that intra adrenal mRNA of key receptors involved in regulating adrenal activity on hormonal synthesis are altered in an animal model of endometriosis and following treatment with a CRHR1 antagonist. This adds support to the hypothesis that there is a dysregulation of the HPA axis during endometriosis progression, which was not completely reversed by pharmacological manipulation with the CRHR1 antagonist antalarmin.

Little is known about intra-adrenal signaling mechanisms. Within the adrenals, mineralocorticoid receptors (MR) bind ACTH to stimulate the production of glucocorticoids. GR, which preferentially bind glucocorticoids are mostly present in the same adrenal cells as MR, and can antagonize one another [7]. Both receptors are steroid receptors of ligand-gated transcription factors, and use cell signaling pathways for non-genomic events [7]. The balance of MR and GR are necessary for homeostatic regulation. Glucocorticoids have more affinity to, and bind to MR at normal levels, while they bind to GR at levels that correspond to those secreted under stress [3].

Aldosterone and cortisol (corticosterone in rats) are ligands that produce the main difference in regulation of MR and GR. MR binds equally to aldosterone, corticosterone and cortisol. However, lower levels of corticosterone cause aldosterone to activate MR and deactivate GR in target cells [7]. While MR mediate electrolyte and fluid homeostasis, GR is responsible for the regulation of energy requirements and decreases inflammatory response [7]. Therefore, the shift in activity towards GR or MR initiated processes is tightly balanced by ligand availability. We observed a decrease in MR in animals with endometriosis at 7 days after the disease initiated (Group 1) and in animals with endometriosis that received antalarmin (Group 2). This suggests that there is a decrease in MR mediated signaling that might favor then GR initiated mechanisms within the adrenals. Our prior study revealed a decrease in endometriosis vesicle number development and size in animals treated with antalarmin. In those same animals, GR receptor mRNA was also increased in endometriotic vesicles compared to normal uterus from the same animal [22]. Taken together, we propose that the decrease in MR is a compensatory mechanism to shift the activity towards GR mediated anti-inflammatory pathways and hence control the endometriosis vesicle development.

**Fig 5:**
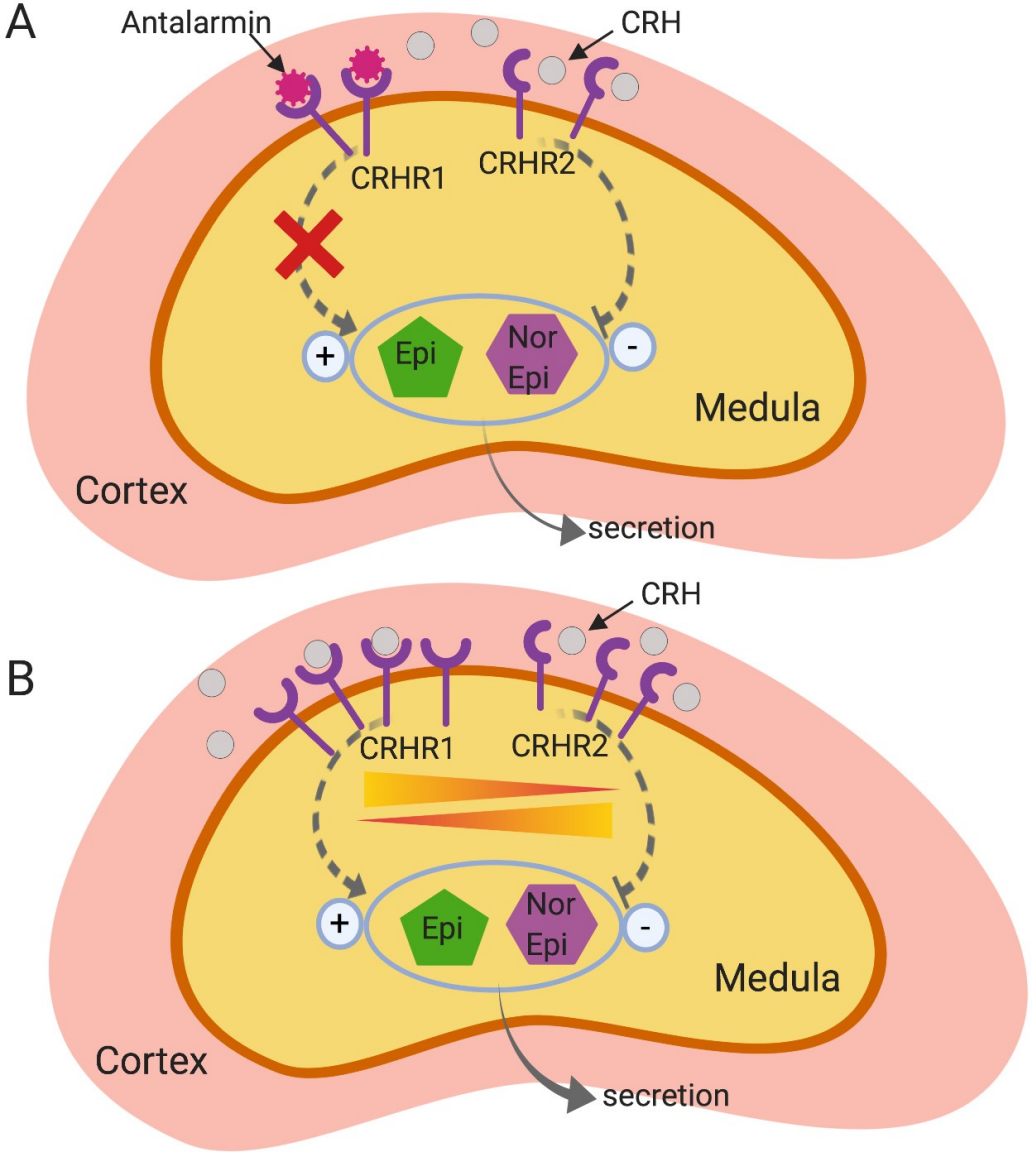
Conceptual diagram of the effects of antalarmin in the adrenal gland. It is known that both CRH receptors are strongly expressed in the medulla of the adrenal gland, which is responsible for the secretion of catecholamines (epinephrine and norepinephrine). **A**: While CRHR1 activation enhances catecholamine release, CRHR2 activation decreases its release. By blocking the CRHR1 receptor with antalarmin for a short period during the initiation of endometriosis, we hypothesize that a decrease in catecholamine release occurs. **B**: When antalarmin was no longer present, there was a reactive increase in CRHR1. However, to counterbalance the increase in CRHR1 activity, we believe that CRHR2 was also upregulated to maintain an appropriate release of catecholamines. CRH: corticotrophin releasing hormone, Epi: epinephrine, NorEpi: norepinephrine. Created with BioRender.com. Figure was exported under a paid subscription.

CRHR1 and CRHR2 are strongly expressed in the rat medulla as well as in the zona glomerulosa of adrenal glands [23]. While CRHR1 agonists produce catecholamine secretion, CRHR2 agonists inhibit catecholamine secretion [23]. Given the fact that we observed an increase in mRNA in both, CRHR1 and CRHR2, in rats with endometriosis that received vehicle, we can infer that catecholamine secretion is also altered due to endometriosis. In fact, we already know that chronic stress exacerbates the progression of endometriosis [24,25] via mechanisms that possibly involve B2 adrenergic receptors [26]. Therefore, an up regulation of CRHR1 signaling within the adrenals might be a compensatory mechanism in response to prior antalarmin administration that is being partially counterbalanced by CRHR2 mRNA increase in order to regulate catecholamine secretion after antalarmin administration (Fig 5).

In this, and both of our previous studies [22,27], sham operated animals served as the controls against which mRNA expression was normalized to measure the effects in rats with endometriosis. Since surgical interventions *per se* pose as a physiological stressor to the animal, comparing against sham groups is an important factor. It is possible that the surgical intervention may alter receptor activity in the adrenals. Unfortunately, we did not have a non-surgical control to measure the expression of receptors in the adrenals. Nevertheless, sham surgery at the same timepoint of the experimental group is the preferred control for surgical effects [28]. It is possible that a longer timepoint between surgery and collection of tissue in group 1 (e.g. 14 or 21 days) may yield a different comparison parameter. In fact, we have previously used a 21-day protocol for sham and endometriosis surgery in studies related to stress and pain perception but did not examine the adrenals in those animals. We believe that the findings in the current manuscript open the door to future experiments.

### Limitations of the study

While similar corticosterone levels in plasma were observed, we did not measure corticosterone binding globulin (CBG, also known as transcortin or SERPINA6). CBG is the major plasma transport protein for corticosterone and progestins. About 78% of corticosterone in plasma is bound to CBG rendering it inactive [29]. CBG has been implicated in chronic inflammatory disorders as well as having a role in fertility outcomes [29,30]. Measurements of corticosterone and cortisol assume normal levels of CBG in plasma but numerous factors such as infections and mutations can change the concentration of CBG [31]. In female rats, CBG has been proposed as a biomarker for inflammation severity [32]. In the clinical scenario, CBG levels in endometriotic tissues have been reported to be lower than in normal endometrium [33], but others have reported normal levels [34]. Hence future animal studies might focus on CBG mediated mechanisms in endometriosis to better elucidate the role of this protein in disease progression.

The autotransplantation rat model of endometriosis has its own limitations. Rats do not develop endometriosis spontaneously. As such, non-human primates might be considered the ideal animal model due to their spontaneous development of endometriosis, similar reproductive physiology and anatomy and phylogenetics [35]. However, the rat model used herein has similar pathological presentations as those observed in women with endometriosis. For example, reduced fertility, impaired NK cell activity, increased vaginal hyperalgesia and a similar global gene expression profile [36–40] have been reported. In addition, ectopic tissue in rats shows similar inflammatory markers, histological characteristics and responds to steroids in a way that resembles ectopic endometrium in women with endometriosis [41–45]. Therefore, the rat model shares many of the hallmark characteristics seen in women with endometriosis (*i*.*e*., subfertility, hyperalgesia, innervation, inflammation), which we believe makes it suitable to the study presented herein.

In summary, we partially reject our original hypothesis postulating that all adrenal receptors will show normal mRNA levels, except for CRHR1. Antalarmin resulted in an increase in CRHR1 mRNA with a concomitant decrease in MCR2. While we cannot differentiate between isolated effects in the adrenal cortex and medulla, the presented work highlights the importance of HPA axis signaling in endometriosis [17,46]. Our study, being the first one exploring intra-adrenal mRNA for receptors involved in the HPA axis as well as in the sympatho-adrenal signaling, calls for additional attention towards adrenal signaling involvement in chronic inflammatory diseases such as endometriosis.
